# Root-associated bacterial community dynamics and assembly mechanisms in healthy and root rot-infected soybeans

**DOI:** 10.3389/fmicb.2026.1789440

**Published:** 2026-04-22

**Authors:** Chunjing Yu, Mengdan Wang, Ming Zhao, Song Zhang, Minghui Cao, Zhiting Liu, Jiaping Jiang, Yi Zhang, Yu Pan, Xiaoyu Zhao

**Affiliations:** Institute of Microbiology, Heilongjiang Academy of Sciences, Harbin, China

**Keywords:** absolute quantitative high-throughput sequencing, community assembly, keystones, rhizosphere soil, soybean root rot

## Abstract

**Introduction:**

Soybean root rot, a devastating soil-borne disease, severely limits global soybean yield and quality. Traditional control measures cause environmental pollution and have regional limitations. Root-associated microbiomes are vital for plant health, but most studies use relative abundance sequencing that distorts actual microbial quantities. This study used absolute quantitative high-throughput sequencing to clarify soil chemical properties and bacterial community assembly in healthy and diseased soybeans, laying a theoretical foundation for microbiome-based root rot management.

**Methods:**

Samples were collected from bulk soil, and the endosphere, rhizoplane, rhizosphere of healthy and diseased soybeans in black soil fields of Heilongjiang. Soil chemical properties, including pH, soil organic carbon (SOC), total nitrogen (TN), available nitrogen (AN), available phosphorus (AP), and available potassium (AK), were determined. Absolute quantitative sequencing of bacterial 16S rRNA V4–V5 region was performed, combined with qPCR for absolute abundance calibration. Bioinformatics analyses included α/β diversity, co-occurrence network, community assembly (βNTI & RCbray), random forest and correlation analysis to identify key taxa and their relationships with environmental factors.

**Results:**

Root rot significantly reduced rhizosphere SOC (by 29.13%), TN (8.57%), AN (24.18%), AP (18.86%), while increased AK (12.82%) and pH. However, the contents of certain bacterial taxa at the genus levels showed significant differences in both absolute and relative abundances. The bacterial co-occurrence network indicate that the interaction in the healthy soybean (H) group was more complex than that in the diseased soybean (S) group. Specifically, 1 module hub and 21 connectors were identified in the H group, whereas 55 connectors were detected in the S group. Community assembly in both the H and S groups was governed by deterministic processes, with homogeneous selection primarily observed in the S group. Random forest and correlation heatmap analyses revealed ASV115 (*Candidatus Koribacter*) in H group was positively correlated with SOC, pH and AN; ASV16 (*Streptomyces*), ASV42 (*Agrobacterium*) and ASV46 (*Mesorhizobium*) were keystones in S group.

**Discussion:**

Root rot destroyed rhizosphere nutrient balance and reshaped bacterial community structure, reducing network complexity. Absolute quantification effectively compensated for the defects of relative abundance, accurately reflecting community changes. These findings clarify the microecological mechanism of soybean root rot, supporting the development of biocontrol strategies targeting rhizosphere microbiome for sustainable soybean production.

## 1 Introduction

Ensuring stable food supply and meeting the growing global demand for food are major challenges for agricultural development in the 21st century (Kopittke et al., 2019). Strengthening integrated disease management and promoting crop health are not only crucial measures for achieving high and stable yields, but also serve as key supports for implementing sustainable agricultural strategies (Beattie et al., 2024; Sharma and Thakur, 2025). Soybean, recognized as one of China’s seven major grain crops and four primary oilseed crops, serves as a crucial source of plant protein, fats, and dietary fiber for both humans and livestock, while playing a vital role in safeguarding national food and oil security (Kumar et al., 2023; Xu et al., 2020). In recent years, although the planting area of soybeans in China has continued to expand, the occurrence and spread of soybean root rot have become one of the key obstacles restricting the improvement of soybean yield (Sun et al., 2023).

Soybean root rot is a global soil-borne disease with strong pathogenic destructiveness and difficulty in prevention and control (Mao et al., 2024). It has become one of the key factors limiting soybean production, and was classified as a Class I crop disease by China’s Ministry of Agriculture and Rural Affairs in 2023 (Yin et al., 2024). The primary fungal pathogens responsible for soybean root rot include *Fusarium* spp. (such as *F. oxysporum*, *F. avenaceum*, *F. solani*, and *F. merismoide*s), *Phytophthora sojae*, and *Pythium ultimum* (Kandel et al., 2023; Xu et al., 2022). This disease in the field is usually caused by the complex infection of multiple different pathogenic bacteria, which also poses greater challenges to the diagnosis and prevention of the disease. Traditional control measures such as seed coating and foliar spraying of chemical fungicides in the field can be used to prevent and control soybean root rot caused by *Fusarium species*, but their limitations have become increasingly prominent (Garud et al., 2024). Chemical fungicides not only pose potential threats to the ecological environment and human health, but also easily induce pathogen resistance, thus severely challenging the sustainability of this control strategy (Dhar Purkayastha et al., 2018; Li et al., 2020). Crop rotation with non-host plants is an effective measure for controlling soybean root rot (Liu Y. et al., 2023). However, its implementation in continuous soybean cropping regions of Northeast China may be limited due to constraints such as scarce arable land, insufficient thermal energy, and the combined effects of specific climatic and economic factors (Deng et al., 2025).

To stabilize soybean yield and promote industrial development, it is imperative to gain an in-depth understanding of the pathogenesis of soybean root rot before implementing targeted prevention and control measures. As the foundation of agricultural ecosystems, soil functional quality is synergistically determined by physical, chemical and biological processes, among which microbial communities play a central pivotal role (Tang et al., 2025). This complex heterogeneous system exhibits the highest level of activity and variability at the rhizosphere interface. Root-associated microbiomes are critical to plant growth and health, and their community composition undergoes dynamic assembly in response to changes in plant developmental stages and soil environmental conditions (Zhou et al., 2025). Plants and microorganisms engage in sustained interactions, establishing a tightly interconnected microenvironment. This dynamic interplay governs the assembly of the root microbiome, commonly referred to as the plant’s “second genome” due to its profound influence on host adaptation and productivity (Abbasi, 2023). The associated soil microenvironment encompasses distinct spatial compartments, including the rhizosphere, rhizoplane and endosphere, all of which serve as key sites for plant–microbe interactions (Bhattacharyya et al., 2021). The rhizosphere microbiota is crucial for maintaining the homeostasis of the rhizosphere microenvironment and safeguarding plant health. Among these microorganisms, there are not only taxa that promote nutrient uptake and transformation, but also those specialized in performing specific functions such as biofilm formation, environmental signal transduction, and intermicrobial recognition (Wang et al., 2022). By virtue of such high functional differentiation, the rhizosphere microbiota can directly or indirectly assist host plants in adapting to and coping with complex and variable environmental stresses (Yang et al., 2024). Rhizoplane microorganisms colonize the root surface and act as a “transit hub” between rhizosphere soil microorganisms and endosphere microorganisms, with their functions mainly reflected in the regulation of interfacial processes (Fan et al., 2025). Endosphere microorganisms colonize the interior of root tissues, establishing a tight symbiotic relationship with plant hosts and jointly forming a functional system characterized by endosymbiosis and precise regulation (Harrison et al., 2021). Therefore, in-depth studies on root-associated microbiomes cannot only provide important evidence for the pathological status of soybean plants, but also guide the formulation of timely and effective biocontrol strategies.

In microbiome research, although relative abundance analysis can reveal variations in community structure, it fails to reflect the actual quantity of each taxon and also cannot directly compare differences in microbial abundance across distinct samples (Bruijning et al., 2023). Studies have demonstrated that estimating microbiome heritability using relative abundance data is subject to multiple limitations, which may induce systematic biases (Wang et al., 2024). First, the interaction dependencies among microorganisms, especially the ecological processes dominated by high-abundance taxa, directly affect the accuracy of heritability estimation. Second, relative abundance analysis tends to generate a high false positive rate under the condition of large sample sizes, leading to an overestimation of the proportion of heritable microbial taxa. In addition, the prevalent co-abundance patterns among microorganisms will further interfere with the reliability of heritability assessment (Azarbad et al., 2022). Current research indicates that the absolute abundance of microorganisms is highly responsive to environmental changes, and can exhibit substantial fluctuations even when their relative abundance remains constant (Wang et al., 2024). Thus, a comprehensive understanding of how the root microbiome assembles and influences plant phenotypes requires a synergistic approach that incorporates quantitative abundance data, which provides substantial research value and scientific insight. Based on this, this study examines the rhizosphere, rhizoplane, and endosphere soils of both healthy and root rot-infected soybean plants, along with bulk soil as a control. By systematically analyzing soil chemical properties and combining absolute quantitative high-throughput sequencing of bacterial communities, we aim to (1) investigate how root rot infection alters the cochemical properties of root-associated soils, (2) clarify the structural, diversity, and absolute abundance changes in key bacterial taxa within the rhizosphere of diseased plants from an absolute quantification perspective, and (3) identify the intrinsic correlations between fluctuations in key soil properties and shifts in bacterial community absolute abundance.

## 2 Materials and methods

### 2.1 Experimental design and root-associated soil sampling

The sampling site was located in an experimental field near the Nenhe Highway in Wudalianchi City, Heilongjiang Province (126°22′ E, 48°49′ N). This region has a cold temperate continental monsoon climate, with a long-term average temperature of 2.1oon climonthly mean temperature drops to as low as −22.7w and the extreme minimum temperature can reach −39an The annual average precipitation is approximately 517 mm, with precipitation concentrated predominantly in summer, accounting for a total of 311 mm. The soil type was classified as black soil, and soybeans were cultivated using a ridge-till system with 65 cm row spacing, following a corn crop. Based on the health status of the soybean plants, samples were collected during the pod-filling stage in August 2024 from the following niches: Bulk soil (CK), endosphere (HE), rhizoplane (HP), and rhizosphere (HR) of healthy soybeans, as well as the endosphere (SE), rhizoplane (SP), and rhizosphere (SR) of soybeans afflicted with root rot. The field sampling adopted a random block design with three independent biological replicates (blocks), and the distance between adjacent blocks was more than 5 m to avoid spatial autocorrelation. In each corresponding plot of the experimental area, five uniformly growing soybean plants of the same treatment (healthy/diseased) were randomly selected. Samples from the same compartment (rhizosphere soil, rhizoplane, and root endosphere) of these five plants were thoroughly mixed to form a single composite sample for subsequent analyses. Bulk soil was collected from the unplanted inter-row area (10 cm away from the soybean ridge, 0–20 cm soil layer) in each block, with 3 composite samples (each mixed from 5 soil cores) as biological replicates (Barberán et al., 2012; Chang et al., 2022).

For the collection of rhizosphere samples, large soil aggregates were gently dislodged from the roots. The soil tightly adhering to the root surface with a thickness of approximately 1–5 mm (defined as rhizosphere soil) was carefully collected using a sterile brush, and the rhizosphere soil from the same treatment in each block was mixed into a composite sample. All rhizosphere soil samples were immediately placed in sterile centrifuge tubes, transported to the laboratory on dry ice, and stored at −80°C for high-throughput sequencing and the assessment of soil chemical properties. To obtain rhizoplane samples, following the rhizosphere soil collection, any remaining soil on the root surface was gently removed using a sterile brush in the laboratory. The roots were subsequently immersed in sterile PBS buffer (pH 7.4) and subjected to sonication in an ultrasonic cleaner (40 kHz, 100 W) for 1 min, followed by centrifugation at 12,000 × g for 1 min. This sonication–centrifugation step was repeated three times. After the final cycle, the supernatant was discarded, and the resulting pellet was collected into centrifuge tubes and stored at −80°C for later sequencing analysis. For endophytic microbial sampling, the roots from which rhizoplane material had been removed were gently dried with sterile absorbent cotton, placed into sterile centrifuge tubes, and stored at −80°C for subsequent analyses, including DNA extraction. The sampling method was based on Bulgarelli et al. (2012).

Soybean root rot was diagnosed by a combination of morphological identification and pathogen isolation and verification: plants with typical root rot symptoms (brown and rotten taproots/lateral roots, reduced root biomass, aboveground stunting, yellowing leaves, and wilting) were initially screened in the field; subsequently, the diseased root tissues were surface-sterilized with 75% ethanol for 30 s and 0.1% HgCl2 for 2 min, rinsed 3 times with sterile water, cut into 0.5 cm small pieces, and cultured on PDA medium at 28ced ro3–5 days. The isolated pathogens were identified as the causal agents of soybean root rot by morphological characteristics and rDNA-ITS sequencing, and only the plants confirmed to be infected by root rot pathogens (*Fusarium* spp., *Phytophthora sojae*, etc.) were defined as diseased plants (S group). Healthy plants (H group) were selected with no visible disease symptoms on roots and aboveground parts, and no pathogenic fungi were isolated from root tissues (Zhang et al., 2023).

### 2.2 Analysis of soil chemical properties

Soil chemical properties were systematically determined, among which the key chemical property was soil pH, and the chemical properties included soil organic carbon (SOC), total nitrogen (TN), alkaline hydrolysable nitrogen (AN), available phosphorus (AP) and fast-acting potassium (AK). Soil pH was measured using a DELTA 320 pH meter (Mettler-Toledo, Switzerland) in a deionized water suspension with a soil-to-water ratio of 1: 2.5 (Begum Raba et al., 2017). SOC was determined using the external heating volumetric method with potassium dichromate and concentrated sulfuric acid (Walkley and Black, 1934). TN in soil was measured using a Kjeldahl nitrogen analyzer after digestion with concentrated sulfuric acid (Page et al., 1982), and the AP content in soil samples was determined using the molybdenum-antimony colorimetric method following extraction with ammonium fluoride-hydrochloric acid (Ekholm and Krogerus, 2003). The content of AK in the soil was measured using the ammonium acetate extraction-flame photometry method (McLean and Watson, 1985). AN in soil was commonly determined by the alkaline hydrolysis diffusion method (Li Y. et al., 2022): the hydrolyzable nitrogen (potentially available nitrogen) was converted into ammonium nitrogen through alkaline hydrolysis, absorbed by a boric acid solution after diffusion, and then titrated with a standard acid. The AN content in the soil was calculated from the titration results.

### 2.3 DNA extraction

Total DNA was isolated from soil and plant root samples using a rapid soil DNA extraction kit (Omega Bio-tek, United States) and a plant DNA extraction kit (Tiangen Biotech, China), respectively, following the manufacturers’ protocols. The concentration and purity of the extracted DNA were determined using a NanoDrop 2000 spectrophotometer (Thermo Fisher Scientific, United States) with the qualified criteria of OD260/280 = 1.8–2.0 and DNA concentration > 20 ng/μL, and DNA integrity was further verified by 1% agarose gel electrophoresis. The qualified DNA was diluted to 10 ng/μL, stored at −80°C until use, and served as the template to amplify the V4-V5 hypervariable region of the bacterial 16S rRNA gene for diversity sequencing library preparation with the primers 341F: 5 -CCTAYGGGRBGCASCAG-3C and 806R: 5 -GGACTACNNGGGTATCTAAT-3GACTACNNGGGTATCTAATsity sequencing library μL reaction system with the program as follows: initial denaturation at 94°C for 5 min, followed by 30 cycles of denaturation at 94°C for 30 s, annealing at 55°C for 30 s and extension at 72°C for 45 s, and a final extension at 72°C for 10 min. Three technical replicates were conducted for each PCR reaction, and the resulting PCR products were pooled and purified using a DNA gel extraction kit (Axygen Biotech, United States).

### 2.4 High-throughput sequencing

For the quantification of bacterial community absolute abundance (expressed as gene copies/g dry soil), absolute quantitative real-time PCR (qPCR) was performed in combination with amplicon sequencing, using the same universal 16S rRNA gene primers (341F/806R) for qPCR amplification. The qPCR amplification program was set as follows: initial denaturation at 94°C for 3 min, followed by 40 cycles of denaturation at 95°C for 15 s, annealing at 62 °C for 20 s, and extension at 72°C for 20 s (Vazyme, Nanjing, China). A standard curve was constructed with plasmid DNA containing the target 16S rRNA gene fragment (Supplementary Figure S3), with the qualified parameters of *R*^2^ > 0.99 and amplification efficiency ranging from 90 to 110%. All qPCR reactions were performed in triplicate on an ABI 7500 Fast Real-Time PCR System to quantify the total 16S rRNA gene copy numbers per gram of dry soil for each sample (Supplementary Figure S1–S3). The relative abundance of each bacterial taxon obtained from high-throughput sequencing was subsequently calibrated by the qPCR-derived total copy numbers and further corrected for 16S rRNA operon copy number bias based on the rrnDB database, and the final absolute abundance of each taxon was expressed as gene copies/g dry soil (Harrison et al., 2021).

Libraries were constructed from the purified PCR amplicons using a TruSeq DNA PCR-Free Library Preparation Kit (Illumina, United States) and quantified with a Qubit 4.0 fluorometer (Thermo Fisher Scientific, United States). Qualified libraries were sent to Lingen Biotechnology Co., Ltd. for paired-end (2 × 300 bp) sequencing on the Illumina MiSeq platform. For each sample, an average of 65,000 raw MiSeq reads were generated, and after quality filtering (removal of low-quality reads with Q < 20, short reads < 200 bp, and chimeric sequences), an average of 60,000 high-quality valid reads were retained for subsequent bioinformatics analysis (Supplementary Table S1).The bioinformatic workflow was as follows: all raw reads were subjected to quality filtering first. Paired-end (PE) reads were merged into single contigs based on overlapping regions (overlap ≥ 10 bp, mismatch rate < 0.2%). Paired reads were trimmed and filtered with a maximum of two expected errors per read (maxEE = 2), then processed using the DADA2 algorithm to resolve amplicon sequence variants (ASVs) by identifying indel mutations and substitutions (Callahan et al., 2016). After merging paired reads and removing chimeric sequences, the taxonomic affiliation of each ASV was assigned using the UCLUST algorithm (Edgar, 2010) against the Silva SSU138.1 16S rRNA database^1^ with a confidence threshold of 80% (Quast et al., 2013). The raw sequencing reads were deposited in the NCBI Sequence Read Archive (SRA) database under Accession Number: SRP674771.

### 2.5 Statistical analyses

Data statistics were performed using Excel, one-way analysis of variance (ANOVA) was conducted using SPSS 25, and multiple comparisons were carried out using Duncan’s multiple range test (α = 0.05). The determination results were expressed as mean ± standard error (SE); graphs were generated using OriginPro 2024b (v10.15). Non-metric multidimensional scaling (NMDS) and multivariate analysis of variance (Adonis), based on Bray-Curtis distance, were performed using the vegan community ecology package (R-forge)^2^ to quantify the contribution of distinct grouping factors to sample differentiation (Kim and Chun, 2014). Co-occurrence network analysis was conducted in R 3.6.3 using the “psych” package based on Spearman’s correlation coefficient (*| r| > 0.7* and *P* < 0.01), and the resulting network data were visualized with Gephi 0.9.2 (Liu et al., 2022). The beta nearest taxon index (βNTI) were computed using the “Picante” package (v1.8.2). The combination of βNTI and Bray-Curtis-based RCbray was used to determine the relative effects of deterministic and stochastic processes on bacterial communities (Xiong et al., 2020). Spearman correlation analysis between keystones and chemical factors was performed using the “Hmisc” packagev (5.1-0) in R 3.6.3 (Liu et al., 2022). Random Forest modeling using the “RandomForest” package (v4.7–1.1) assessed the contribution of individual biomarkers to overall chemical factors and identified the dominant influence variables (Khanum et al., 2023).

## 3 Results

### 3.1 Analysis of chemical properties in root-associated soil

**FIGURE 1:**
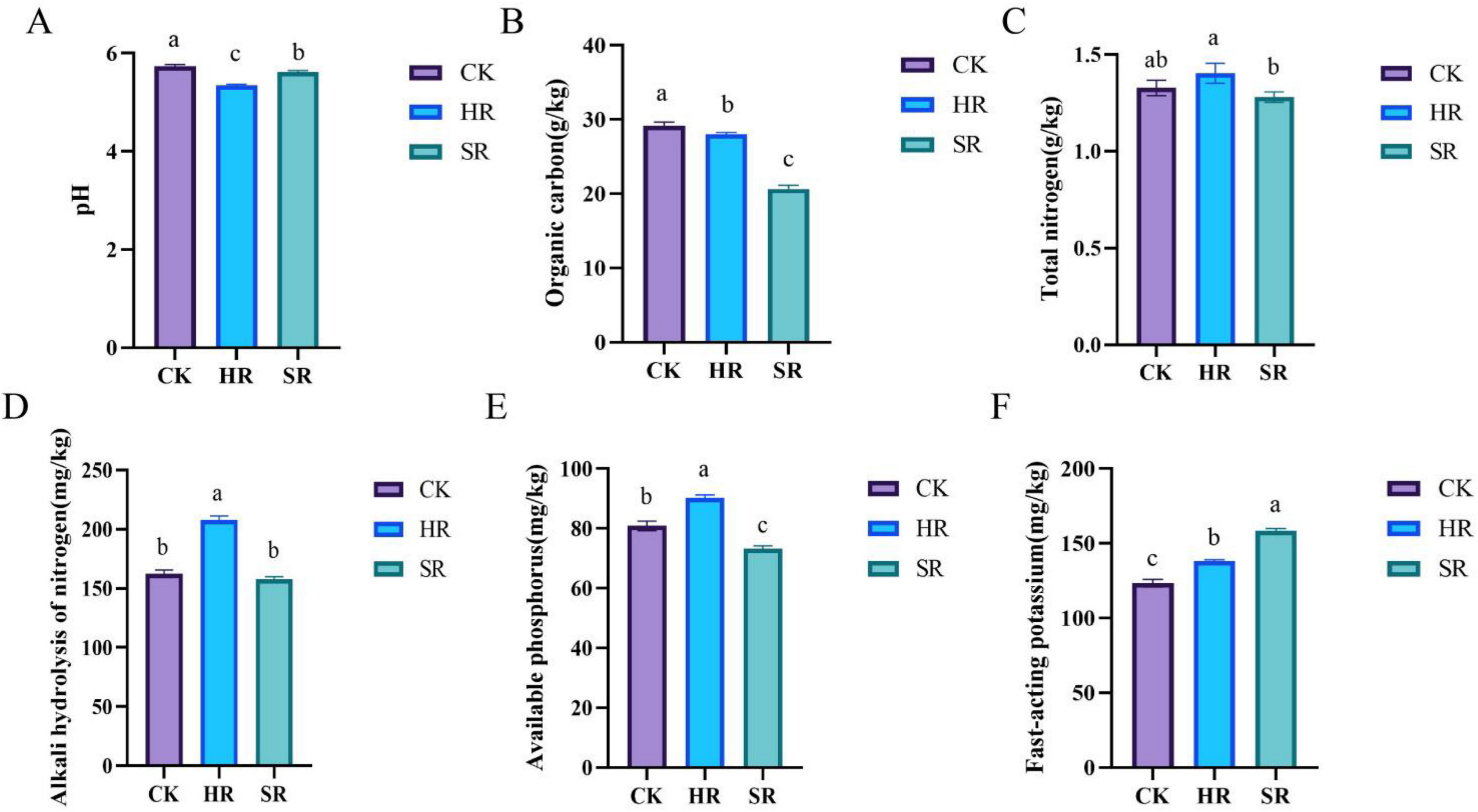
Rhizosphere soil chemical properties under different treatments. **(A)** pH; **(B)** soil organic carbon (SOC); **(C)** total nitrogen (TN); **(D)** alkaline hydrolysis of nitrogen (AN); **(E)** available phosphorus (AP); **(F)** fast-acting potassium (AK). CK, bulk soil from unplanted area; HR, rhizosphere soil of healthy soybean plants; SR, rhizosphere soil of root rot-infected soybean plants. All determinations were performed with three biological replicates (*n* = 3), and data are presented as mean ± standard error (SE). Statistical analyses were conducted using one-way analysis of variance (ANOVA) followed by Duncan’s multiple range test at a significance level of α = 0.05; different lowercase letters above the bars indicate significant differences among treatments (*p* < 0.05).

To reflect the nutrient and environmental characteristics of rhizosphere soil under different planting conditions and plant health statuses, the chemical properties of rhizosphere soil in each treatment group were tested and analyzed. The pH values of the HR, SR, and CK groups in ascending order were 5.34, 5.62, and 5.73, respectively (Figure 1A). The SOC content in the SR group decreased by 29.13%, which was much higher than the 3.87% decrease in the HR group (Figure 1B). Compared with the HR group, the contents of TN, AN, and AP in the SR group decreased by 8.57, 24.18, and 18.86%, respectively (Figures 1C–E). In contrast, the SR group exhibited a 12.82% higher AK content compared to the HR group.

### 3.2 Analysis of bacterial diversity in root-associated soil

**FIGURE 2:**
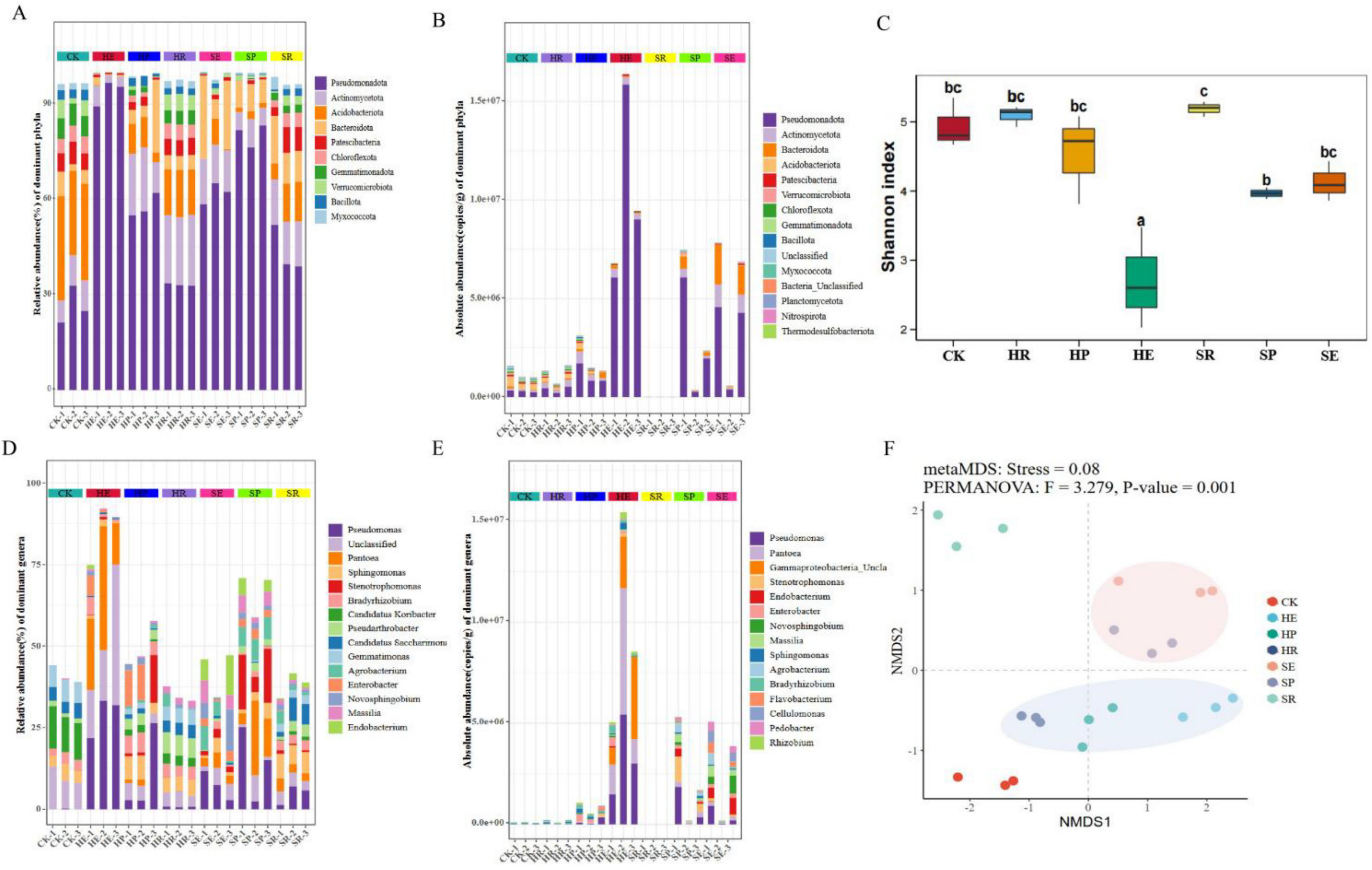
Analysis of bacterial community composition and diversity in root-associated niches. **(A)** Relative abundance of bacterial communities at the phylum level. **(B)** Absolute abundance of bacterial communities at the phylum level. **(C)** Relative abundance of bacterial communities at the genus level. **(D)** Absolute abundance of bacterial communities at the genus level. **(E)** Shannon index (α-diversity) calculated from ASV-level absolute abundance data. **(F)** Non-metric multidimensional scaling (NMDS) based on Bray-Curtis distance of ASV-level absolute abundance data.

The relative and absolute abundances of each treatment group at the phylum level are shown in Figures 2A,B. The results showed that compared with other groups, the relative abundance of *Pseudomonadota* in the CK group was the lowest at 26.32%, while the relative abundance of *Acidobacteriota* was significantly higher than that in other groups, reaching 29.92%. The relative abundances of *Actinomycetota* and *Bacteroidota* were 8.73 and 4.71%, respectively, which reflects the natural community structure of untreated soil. The absolute quantification results at the phylum level showed that *Pseudomonadota* remained the dominant phylum, with its relative proportion reaching 93.82% in the HE group; the relative proportion of *Acidobacteriota* decreased to 6.94%. The HP and HR groups also had relatively high proportions of *Pseudomonadota*, whereas the proportions of *Actinomycetota* and other phyla showed gradient differences. The relative abundance of *Actinomycetota* was higher in HR (21.68%) and HP (16.39%) compared to SE (13.22%), SP (6.71%), and SR (13.95%). In contrast, the proportion of *Acidobacteriota* in HE (6.67%) was significantly lower than that in SE (2.84%), SP (1.81%), and SR (9.87%). The relative and absolute abundances of communities at the genus level are shown in Figures 2C,D. The genera in the CK group were rich and diverse at the relative level, but their absolute abundance was low. In the HE group, *Pseudomonas* (29.01%), *Unclassified* (24.44%), and *Pantoea* (24.20%) exhibited extremely high relative abundances. In contrast, absolute abundance analysis revealed that *Pseudomonas* (28.95%), *Pantoea* (24.17%), and *Gammaproteobacteria_Unclassified* (24.52%) were the dominant genera. The results of SP and SE also indicated differences in the dominant genera between the relative and absolute levels, such as *Candidatus Koribacter* and *Gemmatimonas*.

The α-diversity results (Figure 2E) showed that the Shannon index of the HE group was significantly lower than that of the other groups. There was no significant difference in the bacterial communities between the HR and HP groups and the CK group, but the differences between the SR and SP groups were significant (*p* < 0.05). The β-diversity results of NMDS (Figure 2F) showed that the sample points of the HP and HR groups clustered on the left side of the blue ellipse, and the sample points of the HE group were in the right area, reflecting the structural differentiation within the healthy group, which was consistent with the Shannon index results. The sample points of the SE and SP groups were on the right side of the pink ellipse, and the sample points of the SR group were distributed on the left side of the pink ellipse. Moreover, the soybean root-associated soils from the healthy group and the diseased group showed a clear separated clustering pattern, indicating differences in the rhizosphere bacterial community composition between the diseased and healthy groups.

### 3.3 Bacterial co-occurrence network and topological characterization analysis

**FIGURE 3:**
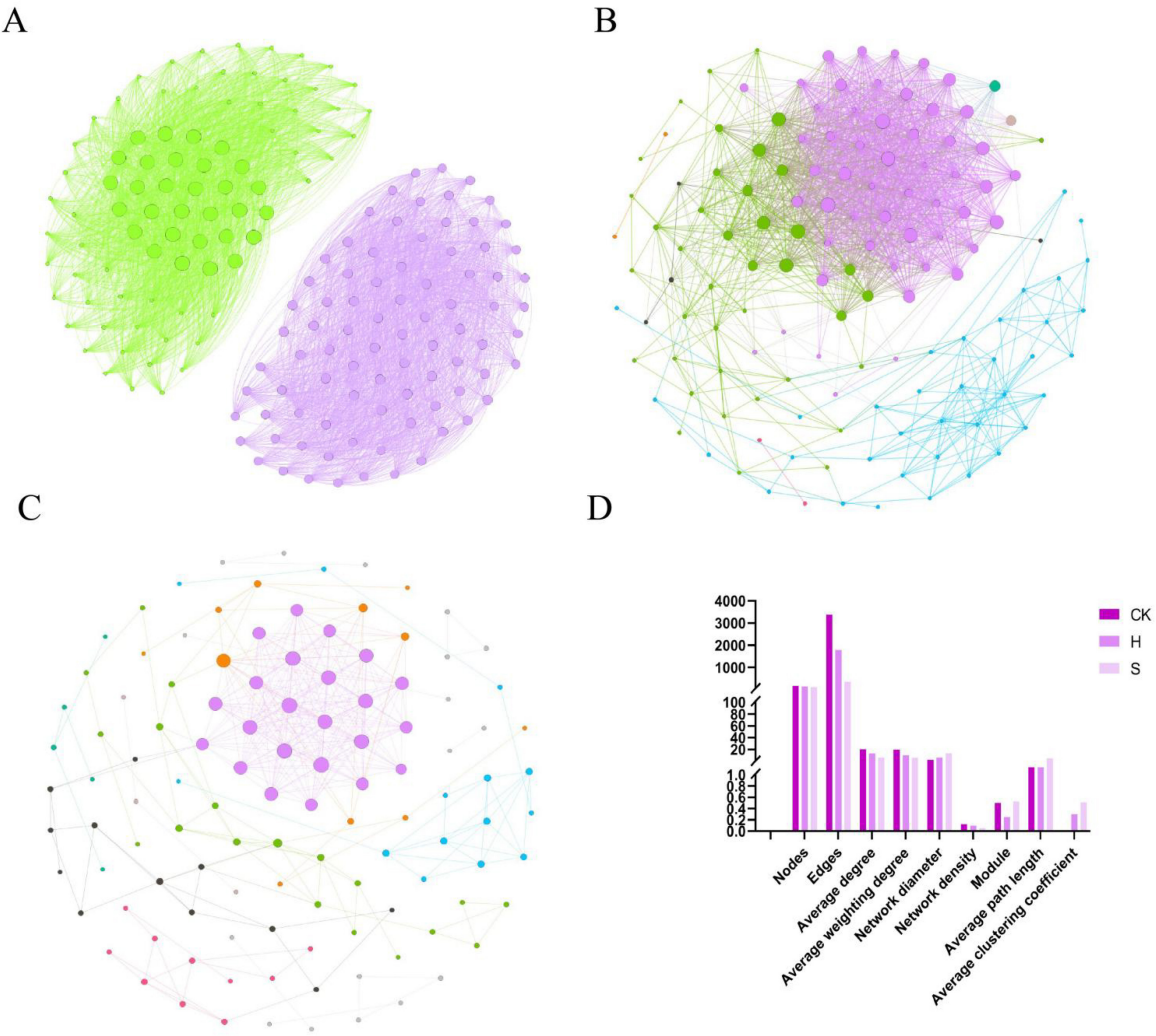
Bacterial co-occurrence network analysis in root-associated niches. **(A)** CK: bulk soil from unplanted area. **(B)** H: the healthy soybean group (HE+HP+HR); **(C)** S: the diseased soybean group (SE+SP+SR); **(D)** the topological characterization of different groups. Nodes represent individual ASVs, with node size scaled by degree. Nodes are colored according to their corresponding core co-occurrence modules. Edges indicate statistically significant correlations (| *r*| > 0.7, *P* < 0.01), and each edge shares the color of the module to which its connected nodes belong.

To evaluate the bacterial interaction patterns in the overall root-associated system of soybeans under different health conditions, a co-occurrence network was constructed at the ASV level using microbial composition data to visualize bacterial interrelationships (Figure 3). The number of edges (3,383) and nodes (168) in the CK group were significantly higher than those in the healthy (H) group (1,788 edges and 138 nodes) and diseased (S) group (354 edges and 114 nodes) (Figures 3A–C). However, only 29.65% of the bacterial correlations in the CK group were positive, which was considerably lower than the 91.61% in the H group and 95.48% in the S group. The average clustering coefficient of the CK group was 0, which was lower than that of the H and S groups. This indicates that although the network of the CK group is more densely connected, the synergistic interactions among bacteria are weaker than those in the other treatment groups, potentially reflecting mutually exclusive relationships as they compete for the same ecological resources. In Figures 3A–C, modules of different colors represent correlated taxonomic clusters identified using a modularity-based approach, reflecting potential functional synergies or niche sharing. Network density indicates the connectivity among nodes within the network, with a higher network density associated with a closer relationship between nodes (Li et al., 2023). The average path length reflects the average distance of information transmission in a network (Zhang et al., 2019). The longer the average path length, the lower the efficiency of information transmission. The results of co-occurrence network analysis showed that the network density of group H was 0.095, which was significantly higher than that of group S (0.055); in contrast, its average path length was 1.924, which was significantly lower than that of group S (4.732) (Figure 3D). This further indicates that the H group had a more connected network and a more complex co-occurrence structure than the S group, which potentially reflects stronger potential synergistic interactions among bacterial taxa in the healthy soybean rhizosphere.

### 3.4 Analysis of keystone taxa and bacterial assembly processes

**FIGURE 4:**
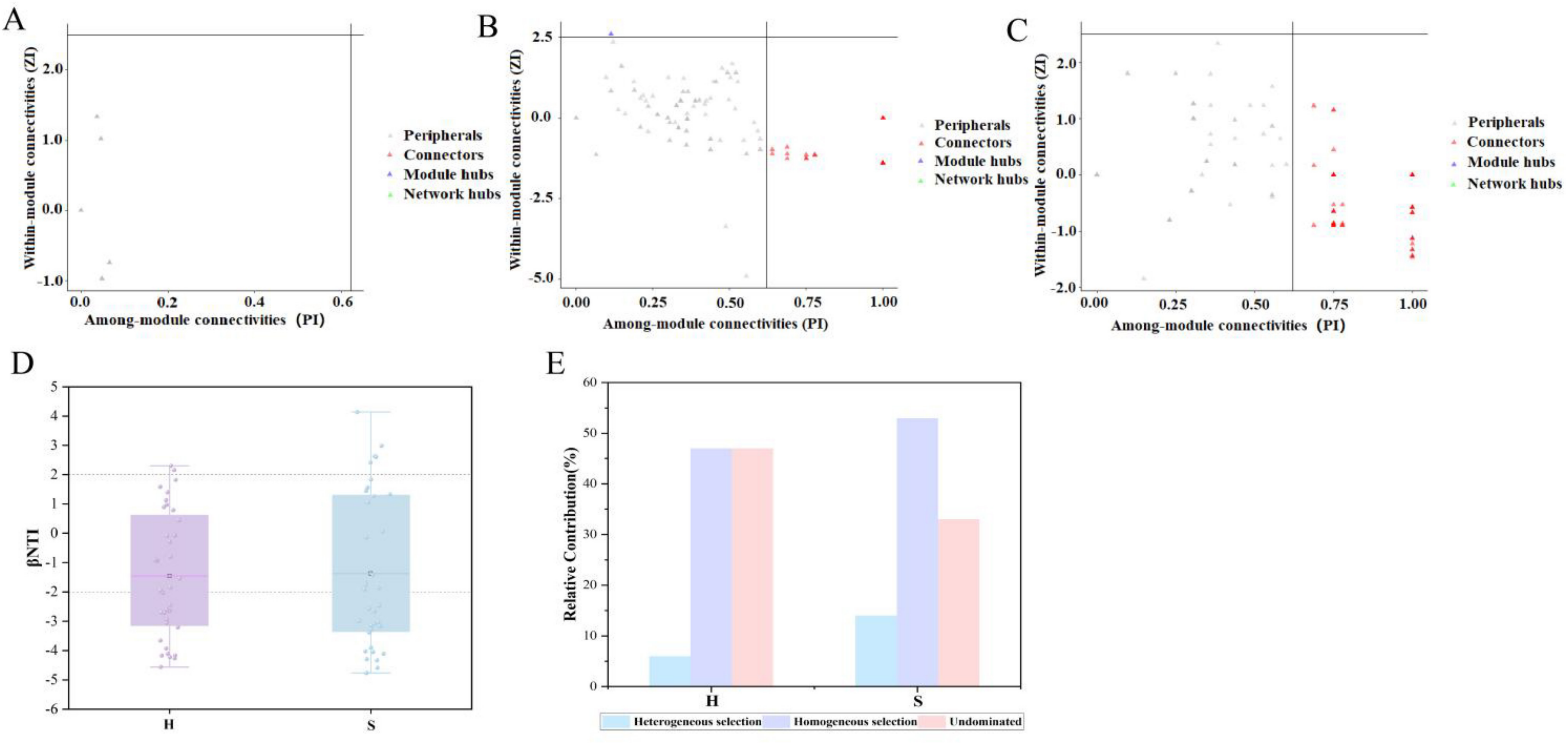
Analysis of keystone species based on ZIPI and bacterial assembly processes. **(A)** CK: bulk soil from unplanted area; **(B)** H: the healthy soybean group (HE+HP+HR); **(C)** S: the diseased soybean group (SE+SP+SR); Node roles were classified using the topological parameters Pi (among-module connectivity) and Zi (within-module connectivity), applying threshold values of 0.62 and 2.5, respectively. **(D)** Distribution of β-nearest taxon index (βNTI) values in the different groups. The dashed line indicates the βNTI threshold (| βNTI| > 2); **(E)** Comparative contribution of deterministic and stochastic processes to community assembly in the different groups.

Based on the previously constructed and validated microbial co-occurrence network, each ASV was assigned a topological role according to its within-module connectivity (Zi) and among-module connectivity (Pi) as follows: peripherals (Zi ≤ 2.5 and Pi ≤ 0.62), module hubs (Zi > 2.5 and Pi ≤ 0.62), connectors (Zi ≤ 2.5 and Pi > 0.62), and network hubs (Zi > 2.5 and Pi > 0.62) (Barberán et al., 2012). In the co-occurrence network of the H group, ASV82 (affiliated with the genus *Novosphingobium* based on taxonomic assignment) served as a module hub (Supplementary Table S3). As shown in Figures 4B,C, the number of connectors in the S group (55) (Supplementary Table S4) was significantly higher than that in the H group (21) (Supplementary Table S2). No keystone were identified in the CK group (Figure 4A).

The results of the ecological process analysis (Figure 4D) revealed significant differences in community assembly mechanisms across different types of rhizosphere soils. Community assembly in the H group was dominated by deterministic processes, accounting for 53%, whereas stochastic processes contributed 47% (Figure 4E). In contrast, the proportion of deterministic processes in the S group was higher, reaching 64%, with stochastic processes accounting for 36% (Figure 4E). Compared to the H group, the S group demonstrated heightened intensities of both the homogeneous and heterogeneous selection.

### 3.5 Biomarker screening and correlations with chemical indices

**FIGURE 5:**
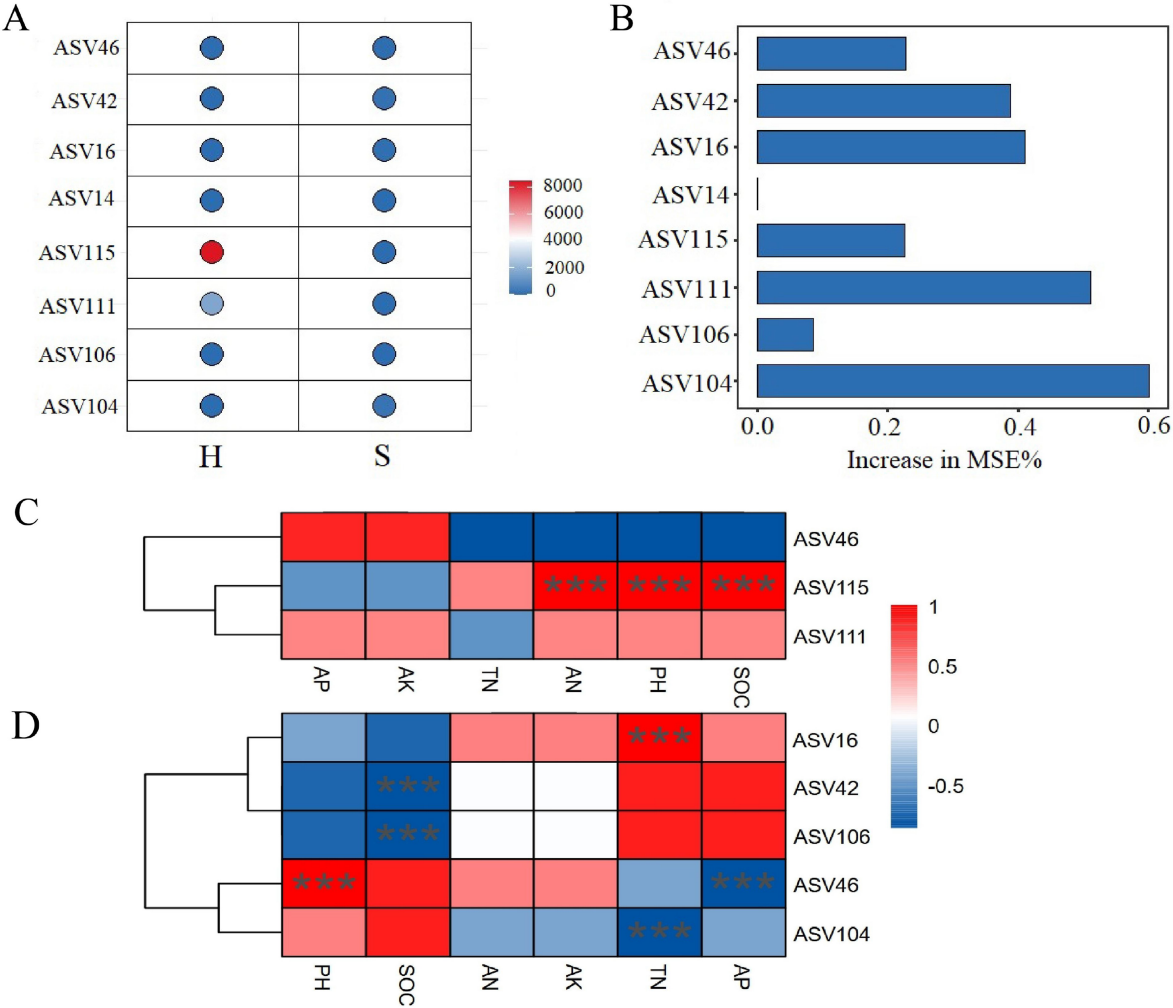
Biomarker screening and correlations with chemical indices. **(A)** Top 8 ASVs identified by random forest analysis in the H and S groups. **(B)** Relative importance of the top 8 ASVs ranked by random forest analysis. Relationship between the top biomarkers and rhizosphere nutrient parameters in **(C)** H group and **(D)** S group. H: the healthy soybean group (HE+HP+HR); S: the diseased soybean group (SE+SP+SR). The taxonomic affiliations of key ASVs are as follows: ASV115 (*Candidatus Koribacter*), ASV16 (*Streptomyces*), ASV42 (*Agrobacterium*), ASV46 (*Mesorhizobium*), ASV104 (*Candidatus Saccharimonas*), ASV106 (*Enterobacter*), ASV111 (*Flavobacterium*), ASV14 (*Pseudoxanthomonas*). ***P < 0.001.

A Random Forest (RF) machine learning algorithm was employed to screen the biomarkers based on the ASVs. Using the Increase in Mean Squared Error percentage (Increase in MSE%) as the importance evaluation metric, the bubble plots of abundance and bar charts of importance for the top eight key ASVs are presented in Figure 5A. ASV115 (assigned to the genus *Candidatus Koribacter*) was significantly more abundant in the H group than in the S group and played a dominant role in the microbial community of healthy root. To further elucidate the relationship between ASV115 and key soil chemical properties, correlation heatmap analysis indicated that its abundance was significantly and positively correlated with SOC, pH, and AN (*p* < 0.01). In the S group, ASV16 (taxonomically assigned to the genus *Streptomyces*) and ASV104 (assigned to the genus *Candidatus Saccharimonas*) were highly significantly correlated with TN (*p* < 0.001). ASV46 (assigned to the genus *Mesorhizobium*) was significantly correlated with both AP and pH. Additionally, ASV42 (assigned to the genus *Agrobacterium*) and ASV106 (assigned to the genus *Enterobacter*) were highly significantly correlated with SOC (*p* < 0.001).

## 4 Discussion

Soybean is a globally important food and cash crop, and its stable production is crucial for safeguarding food security and the agricultural economy (Vargas-Almendra et al., 2024). However, in agricultural production, root rot caused by various soil-borne pathogens (such as *Fusarium* and *Phytophthora*) is one of the major diseases that severely restricts the yield and quality of soybeans (Ren et al., 2024). In this context, the rhizosphere soil, as a dynamic interface for interactions between plant roots and the soil environment, not only directly regulates the availability of water and nutrients but also serves as a critical site for shaping the structure and function of the root microbiome, influencing the competitive balance between pathogenic and beneficial microbial communities. Soil physicochemical properties constitute the core foundation for maintaining the functional stability of agro-ecosystems and achieving high crop yield and quality (Chaudhry et al., 2024). Particularly in the rhizosphere, favorable physicochemical conditions can facilitate the assembly of beneficial microbial communities, inhibit the propagation of soil-borne pathogenic microorganisms, and directly regulate root system development and stress resistance.

Compared with the CK group, the pH values of HR and SR groups are significantly lower (*p* < 0.05) (Figure 1A). This may be because soybean cultivation enhances the metabolic activity of soil bacteria, and plant-microbe interactions alter the material transformation process, resulting in changes in the pH value of the rhizosphere microenvironment (Fan et al., 2025). The transformation of organic carbon, nitrogen, phosphorus, and potassium into plant-available forms via microbial mineralization and decomposition represents a key nutrient acquisition route for terrestrial plants (Jiao et al., 2022). As shown in Figure 1B, the SOC contents of the different groups were in the order of CK > HR > SR, indicating that microorganisms in the HR and SR groups have effectively utilized and transformed SOC. Specifically, microorganisms in HR group may decompose and utilize SOC more actively to provide energy for their own metabolism and nutrient transformation. The lowest SOC content in the S group is likely attributable to severely impaired plant roots due to pathogen infection, which drastically reduces carbon sources (e.g., root exudates) secreted into the rhizosphere, while soil-borne organic compounds are directly utilized as nutrients by root rot pathogens such as *Fusarium* spp., thereby accelerating the depletion of SOC. The TN content in both the HR and SR groups was not significantly different from that in the CK group; however, the TN content in the HR group was significantly higher than that in the SR group (*p* < 0.05) (Figure 1C). The AN content of HR is significantly higher than those of CK and SR groups (*p* < 0.01) (Figure 1D). These results suggest that rhizosphere microorganisms of healthy plants may play a stronger role in nitrogen fixation or promoting organic nitrogen accumulation, thus maintaining high TN and AN levels (Chang et al., 2022). However, in the SR group, the reduction in TN reserves and the decrease in AN supply may be attributed to the weakened nitrogen-fixing capacity of rhizosphere microorganisms under disease conditions (Liu W. et al., 2023). The AP content in the HR group was significantly higher than that in the CK and SR groups (*p* < 0.01) (Figure 1E), indicating that the bacterial community in the healthy soybean rhizosphere may regulate soil phosphorus bioavailability through multiple potential pathways, with the specific mechanisms to be further verified by functional trait determination (Li H. et al., 2022). Compared with the CK group, the AK content in the SR group was significantly higher than in the HR and CK groups (Figure 1F). This indicates that within the SR group, alterations in the microbial community structure or biological activity may have led to either aberrantly vigorous activity of potassium-solubilizing microorganisms or a reduction in potassium uptake by plants, consequently resulting in AP accumulation (Liu et al., 2021). These differences imply that the growth and metabolism of pathogenic bacteria may produce alkaline substances, thereby affecting nutrient availability. The rhizosphere is not only the core area for the occurrence of soil-borne diseases but also a hotspot for interactions among microorganisms, pathogens, and plants (Escudero-Martinez et al., 2022). The structural and functional characteristics of the microbial community in this region play a key regulatory role in the occurrence and development of disease.

As shown in the results of Figures 2A–D, there were significant differences in the composition and diversity of bacterial communities at the phylum and genus levels among the different groups. This phenomenon indicates that during the growth and development process, plants not only enhance their own microbial carrying capacity but also strengthen the selective pressure on rhizosphere microorganisms (Montes de Oca-Vásquez et al., 2020). The formation of such selective effects is largely derived from the interaction effects during the long-term co-evolution of plants and microorganisms. However, the contents of certain bacterial species varied considerably in terms of both absolute and relative levels. Currently, most studies on soil microbial communities employ 16S rRNA gene sequencing for relative quantification of microbial diversity to conduct comparative analyses. This approach lacks correction for microbial biomass, which may lead to misjudgments when interpreting the roles of microbial populations (Ren et al., 2025). If only relative abundance and phenotypic traits are used for correlation analysis, it will affect the correlation analysis between microbial community characteristics and quantitative data (e.g., physiological parameters), resulting in misjudgment of conclusions. Analysis of the relative and absolute abundances of key functional bacteria demonstrated that species composition differences indicated by relative abundance do not necessarily accurately reflect the actual variation trends in microbial absolute abundance—*Candidatus Koribacter* and *Gemmomonas* are typical examples. The similar bacterial levels observed in bulk and rhizosphere soils in Figure 2B, E may arise from a combination of multiple factors; notably, although the rhizosphere effect was not manifested in terms of microbial abundance in this study, the rhizosphere bacterial community exhibited distinct structural and functional differentiation from that of bulk soil (e.g., evident in NMDS clustering and the enrichment of nutrient cycling-related taxa), which represents the core manifestation of the rhizosphere effect in our research.

The co-occurrence network analysis (Figure 3) showed that the microbial network of the CK group exhibited significantly greater size and complexity compared to the H and S groups. However, the proportion of positive interactions within its network was substantially lower than that in the other two groups. This is likely due to the greater stability of bacterial communities in non-soybean soil, with no soybean root exudate-induced selective enrichment or rhizosphere-specific interactive taxa present (Du et al., 2022). Prior investigations have established that elevated positive correlations within microbial networks facilitate inter-microbial interactions while attenuating competitive pressures, and that these interactional dynamics exert a more direct regulatory role in sustaining community stability relative to microbial diversity (Chen et al., 2025). Network topological characteristics indicate that the H group possesses higher network complexity than the S group. Studies have shown that the invasion of root rot pathogens such as *Fusarium* inherently disrupts the original balance of microbial interactions. When these pathogens become keystone taxa or are significantly enriched in the co-occurrence network, they may potentially alter the inferred resource flow and interspecific co-occurrence relationships, leading to the disintegration and simplification of the original complex co-occurrence network, thereby potentially weakening the disease resistance potential of the rhizosphere microecosystem (Zhang et al., 2025).

In the co-occurrence network of the H group, ASV82 (identified as *Novosphingobium*) served as a module hub (Figure 4B). By forming close metabolic associations within its module, this taxon likely effectively occupies a specific ecological niche and gains a competitive advantage over coexisting taxa with overlapping niches (Wang et al., 2025). Furthermore, its high connectivity may have helped buffer environmental fluctuations, thereby contributing to the maintenance of metabolic stability (Woźniak et al., 2019). Research indicates that *Novosphingobium* can produce phytohormones, such as indole-3-acetic acid, supporting plant growth and stress resistance through mutualistic interactions (Ma et al., 2020). In natural environments, the core ecological functions of microorganisms often require coordinated efforts across multiple functional modules, with connectors playing a key role in enabling such cross-module functional integration (Li et al., 2025). As shown in Figures 4B,C, the number of connectors in the S group (55) was significantly higher than that in the H group (21). In healthy rhizospheres, the microbial community primarily serves the singular objective of promoting soybean growth, which entails simpler inter-module cooperation. In contrast, diseased rhizospheres require the concurrent functions of growth promotion and disease resistance. This dual functional demand drives the enrichment of dual-function connectors, which enhance cross-module interactions to maintain functional stability (Blagodatskaya and Kuzyakov, 2013). No keystone were identified in the CK group (Figure 4A). Compared to the rhizosphere soil in planted zones, the absence of root exudates (such as sugars and amino acids) in unplanted areas results in a lack of specialized carbon sources, leading to reduced metabolic activity of microbial communities and limiting them to the decomposition of basic organic matter. Under these conditions, microbial biomass is lower, activity is weakened, and the microbial network becomes simpler and more loosely connected, thereby preventing the formation of highly connected keystones (Yang et al., 2022).

Deterministic processes (such as environmental filtering and host-specific interactions) play a dominant regulatory role in the assembly of rhizosphere soil microbial communities. These processes are collectively driven by factors including soil pH, nutrient availability, and plant species, which can exert a selective enrichment effect on specific microbial taxa. In contrast, stochastic processes (such as random dispersal and ecological drift) have a weaker regulatory impact on the structure of rhizosphere microbial communities (Yuan et al., 2023). The S group demonstrated higher intensities of both homogeneous and heterogeneous selection than the H group (Figures 4D,E). Disease-induced systemic stress in soybean plants causes rhizosphere dysbiosis. This common stress acts as an environmental filter, deterministically selecting specific microbial groups adept at pathogen competition, thus increasing homogeneous selection (Zhang et al., 2023). Furthermore, the emergence of localized root necrosis, decay, and loss of functional compartmentalization in diseased plants creates pronounced microenvironmental variations. Each distinct niche filters for an adapted microbial community, driving community divergence and resulting in an increase in heterogeneous selection (Yin et al., 2021). Random forest analysis was employed to screen biomarkers and validate their associations with rhizosphere soil chemical factors (Figure 5). Compared with the H group, the S group contained more core ASVs that were significantly correlated with chemical indicators, suggesting that root rot pathogen invasion may have activated certain indigenous microorganisms to actively engage in nutrient transformation and utilization (Yin et al., 2021).

**FIGURE 6:**
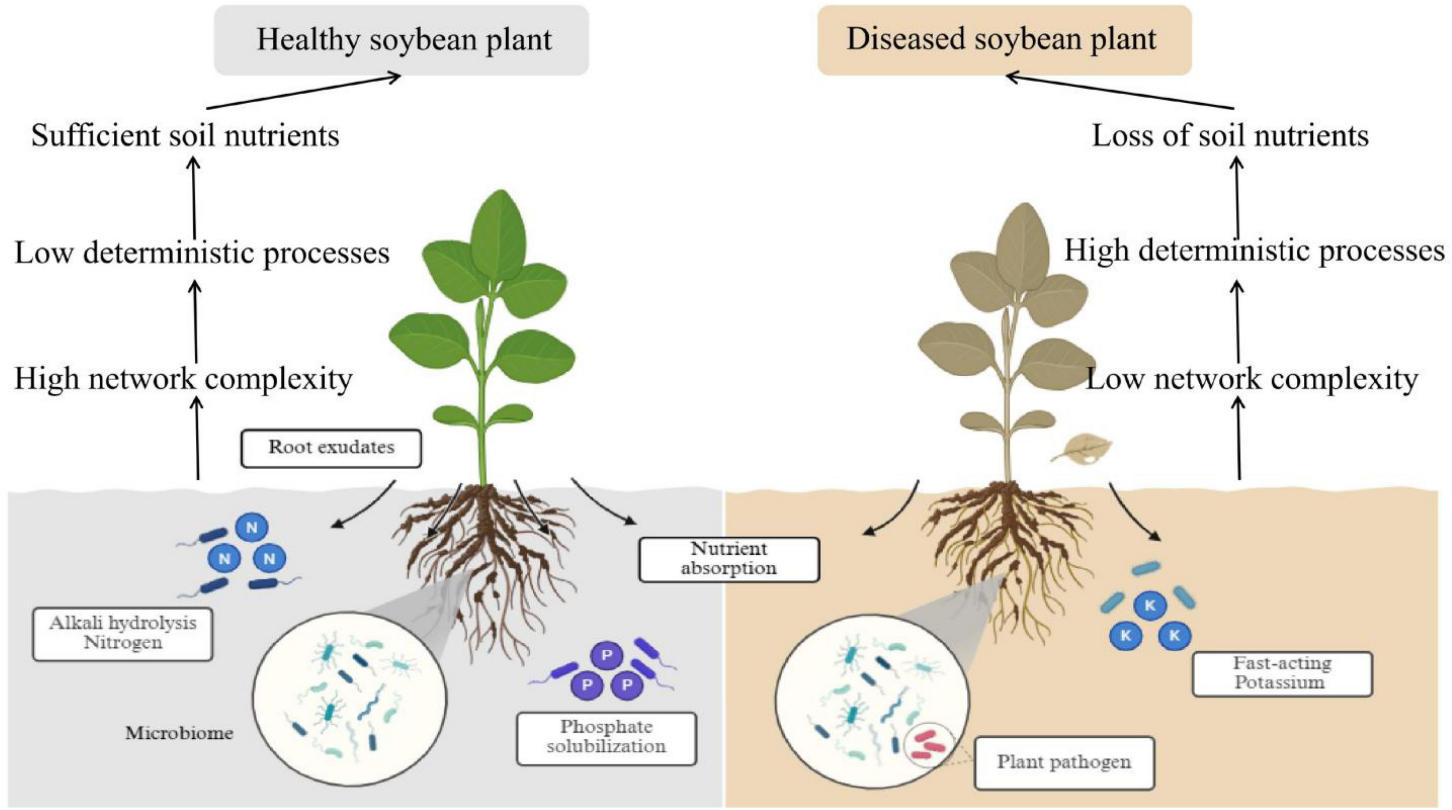
Conceptual model depicting bacterial community dynamics and their link to nutrient transformation processes in healthy and diseased soybean roots. Created in BioRender. Yu, C. (2026) https://BioRender.com/u8rfct9.

To elucidate the differences in the rhizosphere microecology between healthy and diseased soybean plants, this study constructed a corresponding mechanism diagram (Figure 6). In the H group, ASV115 was identified as a key species, and its relative abundance showed significant positive correlations with SOC, pH, and AN (Figure 5C). ASV115 belongs to the genus *Candidatus Koribacter*, which includes taxa involved in soil nitrogen cycle processes such as ammonification and auxiliary nitrification, thereby contributing to the accumulation of available nitrogen (Vargas-Almendra et al., 2024). Thus, we hypothesize that ASV115 may promote soybean health by participating in the regulation of carbon and nitrogen cycling and helping to maintain the chemical stability of the rhizosphere soil in healthy soybean plants. In the S group, ASV16 (assigned to the genus *Streptomyces*), ASV42 (assigned to the genus *Agrobacterium*), and ASV46 (assigned to the genus *Mesorhizobium*) were simultaneously screened as keystone by both Zi-Pi analysis and the random forest model. This indicates that under the disturbance of root rot pathogens, even though the interactions between soybean roots and microorganisms tend to become simplified, these computationally identified keystone species may potentially affect the rhizosphere microecology of diseased soybean plants by enhancing deterministic assembly processes, and their actual functional roles need to be further verified by experimental validation.

This study focused on the dynamic changes in root-associated bacterial communities and rhizosphere soil chemical properties between healthy and root rot-infected soybeans, and revealed the potential regulatory mechanisms of the microbiome on plant health. However, direct measurements of plant performance traits were not included in the present study, which limits the direct quantification of the link between microbiome shifts and soybean growth and yield losses induced by root rot. In addition, although we conducted a semi-quantitative assessment of root rot disease severity, the lack of continuous dynamic monitoring of disease development and plant growth stages hinders a more in-depth analysis of the causal relationship between microbial community assembly and the progression of soybean root rot. Future studies will combine continuous phenotyping of soybean growth, yield components, and dynamic disease severity monitoring with multi-omics approaches (e.g., metabolomics, transcriptomics) to further validate the functional roles of keystone taxa in regulating soybean health and yield formation.

## 5 Conclusion

This research delivers unique insights into the assembly mechanisms and potential functions of soybean root-associated microorganisms under different conditions. Our findings demonstrate that root rot significantly reshapes the chemical properties of soybean rhizosphere soil, reducing SOC content and the availability of nitrogen and phosphorus while increasing the level of AK. This drives the reconstruction of the rhizobacterial community structure, with decreased absolute abundance and complexity of the co-occurrence network. Meanwhile, ASV16 (assigned to the genus *Streptomyces*), ASV42 (assigned to the genus *Agrobacterium*), and ASV46 (assigned to the genus *Mesorhizobium*) may act as keystone species by reinforcing deterministic processes to adapt to the rhizosphere microenvironment under the disturbance of root rot pathogens. The dynamic changes in the rhizosphere generalist core microbiome can partially explain the variations in plant traits. The above findings lay a solid foundation for future research and application of biological control against soybean root rot based on soil microecological regulation. However, the conclusions of this study are primarily based on correlations between rhizosphere bacterial community shifts and soil chemical properties; the absence of direct plant performance measurements (e.g., biomass, yield) limits a direct evaluation of the actual agricultural impacts of these microbial changes. Future in-situ field experiments incorporating systematic phenotyping will further verify the functional significance of key microbial taxa and their practical value in soybean production.

## Data Availability

The original contributions presented in the study are included in the article/Supplementary material, further inquiries can be directed to the corresponding authors.

## Footnotes

1
http://www.arb-silva.de

2
https://r-forge.r-project.org/
