# Supplementary material for: Root-associated bacterial community dynamics and assembly mechanisms in healthy and root rot-infected soybeans
Source: Front Microbiol. 2026 Apr 22;17:1789440. doi: 10.3389/fmicb.2026.1789440 (PMC13144118; doi:10.3389/fmicb.2026.1789440)
Supplement: Supplementary file 1 [file Supplementary_file_1.docx]

*Root-associated Bacterial Community Dynamics and Assembly Mechanisms in Healthy and Root Rot-infected Soybeans*

Chunjing Yu^1†^ , Mengdan Wang ^1†^ , Ming Zhao^1^, Song Zhang^1^ , Minghui Cao^1^ , Zhiting Liu^1^ , Jiaping Jiang^1^ , Yi Zhang^1^ , Yu Pan ^1,^*, Xiaoyu Zhao^1,^*

1 Institute of Microbiology, Heilongjiang Academy of Sciences, Harbin 150000, [China](mailto:China;yu0815004@163.com(C.Y.);)

† These authors have contributed equally to this work

* Correspondence: [zhao1612@163.com;](mailto:236183617@qq.com;) [panyu198701@163.com](mailto:panyu198701@163.com)


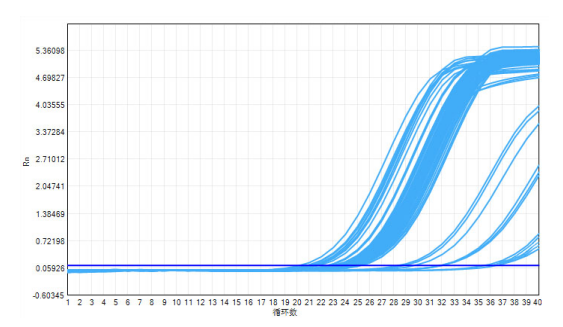

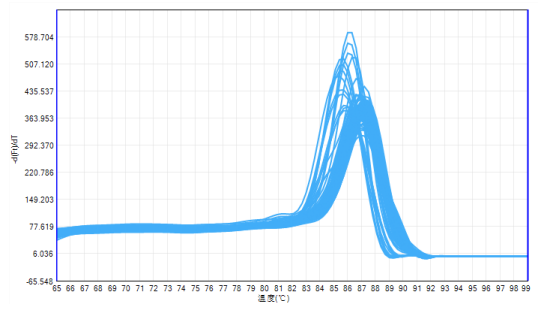


A

B

Figure S1 The fluorescence quantitative PCR amplification curve (A) and melting curve (B) of the gene sample

Figure S2 The fluorescence quantitative PCR amplification curve (A) and melting curve (B) of the gene standard sample


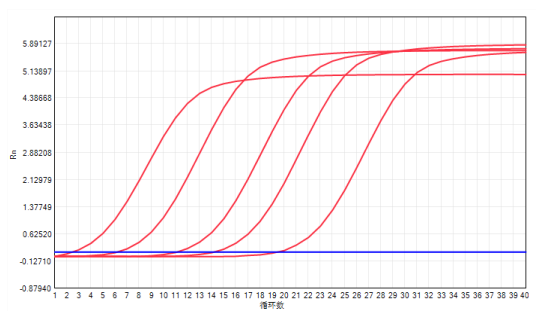

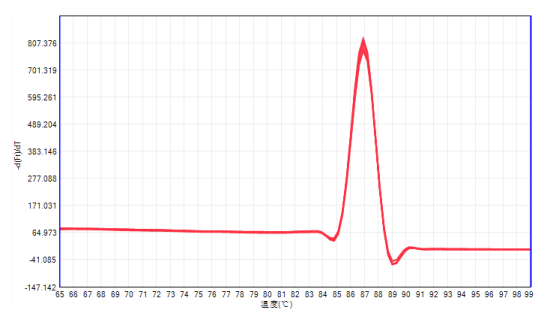


A

B


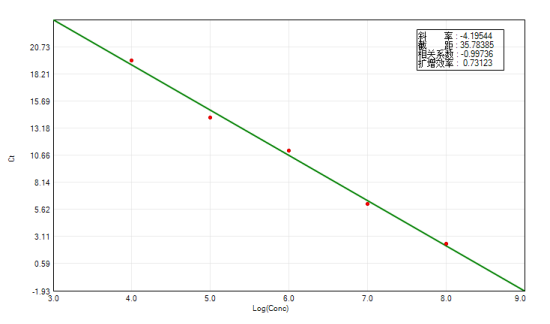


Figure S3 Genetic standard curve

Table S1 Effective sequence statistics

| Samples | Reads | Bases(bp) | AverageLength(bp) |
| --- | --- | --- | --- |
| CK1 | 72422 | 14923808 | 412.13 |
| CK2 | 60372 | 12386346 | 410.33 |
| CK3 | 60556 | 12491156 | 412.55 |
| HE1 | 71026 | 14932638 | 420.48 |
| HE2 | 74440 | 15750709 | 423.18 |
| HE3 | 70292 | 14666726 | 417.31 |
| HP1 | 60536 | 12554177 | 414.77 |
| HP2 | 66022 | 13663503 | 413.91 |
| HP3 | 61160 | 12872602 | 420.95 |
| HR1 | 70282 | 14545668 | 413.92 |
| HR2 | 73252 | 15167949 | 414.13 |
| HR3 | 69790 | 14453237 | 414.19 |
| SE1 | 69254 | 14455961 | 417.48 |
| SE2 | 72586 | 15101572 | 416.1 |
| SE3 | 70488 | 14611704 | 414.59 |
| SP1 | 66424 | 13991610 | 421.28 |
| SP2 | 74180 | 15467301 | 417.02 |
| SP3 | 65766 | 13818953 | 420.25 |
| SR1 | 61940 | 12855885 | 415.11 |
| SR2 | 60166 | 12461977 | 414.25 |
| SR3 | 60614 | 12536981 | 413.67 |

Table S2 Number of connectors in the Zi-Pi results of the healthy group

| **Numbers** | **nodes_id** | **within_module_connectivities** | **among_module_connectivities** |
| --- | --- | --- | --- |
| 1 | ASV51 | -1.149 | 0.778 |
| 2 | ASV89 | -1.149 | 0.778 |
| 3 | ASV343 | -1.122 | 0.639 |
| 4 | ASV7 | -0.982 | 0.639 |
| 5 | ASV19 | -1.142 | 0.066 |
| 6 | ASV26 | -1.142 | 0.066 |
| 7 | ASV120 | -1.399 | 1 |
| 8 | ASV1213 | -1.402 | 1 |
| 9 | ASV282 | -1.399 | 1 |
| 10 | ASV285 | 0 | 1 |
| 11 | ASV348 | -1.402 | 1 |
| 12 | ASV354 | 0 | 1 |
| 13 | ASV703 | -1.399 | 1 |
| 14 | ASV827 | 0 | 1 |
| 15 | ASV854 | 0 | 1 |
| 16 | ASV299 | -1.262 | 0.75 |
| 17 | ASV475 | -1.262 | 0.75 |
| 18 | ASV9 | -1.149 | 0.75 |
| 19 | ASV1 | -1.122 | 0.6875 |
| 20 | ASV166 | -1.262 | 0.6875 |
| 21 | ASV194 | -0.899 | 0.6875 |

Table S3 Number of module hubs in the Zi-Pi results of the healthy group

| **Numbers** | **nodes_id** | **within_module_connectivities** | **among_module_connectivities** |
| --- | --- | --- | --- |
| 1 | ASV82 | 2.607 | 0.114 |

Table S4 Number of connectors in the Zi-Pi results of the disease group

| **Numbers** | **nodes_id** | **within_module_connectivities** | **among_module_connectivities** |
| --- | --- | --- | --- |
| 1 | ASV108 | -0.651 | 0.75 |
| 2 | ASV1136 | 0 | 0.75 |
| 3 | ASV117 | 0 | 0.75 |
| 4 | ASV1336 | -0.577 | 1 |
| 5 | ASV136 | -0.535 | 0.75 |
| 6 | ASV147 | 0 | 1 |
| 7 | ASV15 | -0.577 | 1 |
| 8 | ASV156 | -0.577 | 1 |
| 9 | ASV16 | -0.897 | 0.778 |
| 10 | ASV17 | -0.897 | 0.75 |
| 11 | ASV1735 | 0 | 1 |
| 12 | ASV174 | 1.225 | 0.688 |
| 13 | ASV180 | -0.651 | 0.75 |
| 14 | ASV187 | 0 | 1 |
| 15 | ASV188 | -1.123 | 1 |
| 16 | ASV19 | -1.333 | 1 |
| 17 | ASV191 | 0.447 | 0.75 |
| 18 | ASV206 | -1.333 | 1 |
| 19 | ASV221 | 0 | 1 |
| 20 | ASV225 | 0 | 0.75 |
| 21 | ASV226 | -0.535 | 0.778 |
| 22 | ASV229 | 0 | 0.75 |
| 23 | ASV24 | -0.897 | 0.75 |
| 24 | ASV248 | -1.225 | 1 |
| 25 | ASV256 | -1.436 | 1 |
| 26 | ASV27 | -0.671 | 1 |
| 27 | ASV3 | -0.897 | 0.75 |
| 28 | ASV311 | -0.671 | 1 |
| 29 | ASV330 | -0.867 | 0.75 |
| 30 | ASV358 | 0.163 | 0.688 |
| 31 | ASV362 | 1.155 | 0.75 |
| 32 | ASV366 | -1.464 | 1 |
| 33 | ASV412 | -0.867 | 0.75 |
| 34 | ASV42 | -1.123 | 1 |
| 35 | ASV430 | -1.436 | 1 |
| 36 | ASV431 | 0 | 0.75 |
| 37 | ASV46 | -0.866 | 0.778 |
| 38 | ASV50 | -0.651 | 0.75 |
| 39 | ASV504 | 0 | 1 |
| 40 | ASV514 | 0 | 1 |
| 41 | ASV533 | -0.577 | 1 |
| 42 | ASV55 | 0 | 0.75 |
| 43 | ASV57 | -0.897 | 0.75 |
| 44 | ASV587 | -0.671 | 1 |
| 45 | ASV6 | 0 | 1 |
| 46 | ASV603 | -0.897 | 0.688 |
| 47 | ASV679 | 0 | 0.75 |
| 48 | ASV7 | -0.651 | 0.75 |
| 49 | ASV74 | -0.897 | 0.778 |
| 50 | ASV87 | 1.155 | 0.75 |
| 51 | ASV89 | -1.123 | 1 |
| 52 | ASV92 | -0.866 | 0.75 |
| 53 | ASV929 | -0.867 | 0.75 |
| 54 | ASV95 | -0.867 | 0.75 |
| 55 | ASV97 | 0 | 1 |
